# Daily care hours among older emergency department patients with dementia and undiagnosed cognitive impairment: a cross‐sectional study

**DOI:** 10.1002/alz.084065

**Published:** 2025-01-09

**Authors:** James Galske, Tonya Chera, Ula Hwang, Joan Monin, Arjun Venkatesh, Kenneth Lam, Amanda N Leggett, Cameron Gettel

**Affiliations:** ^1^ University of Connecticut School of Medicine, Farmington, CT USA; ^2^ Yale School of Medicine, New Haven, CT USA; ^3^ NYU Langone Health, New York, NY USA; ^4^ Yale School of Public Health, New Haven, CT USA; ^5^ University of California San Francisco, San Francisco, CA USA; ^6^ Wayne State University, Detroit, MI USA

## Abstract

**Background:**

Over 15 million informal caregivers provide assistance to persons living with dementia. Despite increasing emergency department (ED) use within the population, little is known regarding the support required of older adults seeking acute care with varying degrees of cognitive impairment. Our objectives were to quantify the daily care hours that informal caregivers provide to older ED patients with diagnosed dementia, undiagnosed cognitive impairment, and intact cognition.

**Methods:**

We conducted a cross‐sectional analysis of caregivers of community‐dwelling older adults aged 65+ years seeking emergency care. The caregiver‐completedAD8 (cAD8) was administered to screen for cognitive impairment among caregivers of older ED patients without a diagnosis of dementia indicated in the electronic health record. Based on the caregiver’s response, older adults were categorized into groups of: diagnosed dementia, undiagnosed cognitive impairment (cAD8 score of 2+), and cognitively intact (cAD8 score of <2). The primary outcome was mean self‐reported hours of care per day provided by the caregiver assessed using the Mann‐Whitney U test.

**Results:**

Our analytic sample included caregivers of 439 older ED patients; 58 had diagnosed dementia, 69 had undiagnosed cognitive impairment, and 312 had intact cognition. Hours of care per day provided by caregivers were significantly greater for older adults with diagnosed dementia (13.8 hours, P<0.0001) and undiagnosed cognitive impairment (9.9 hours, P<0.0001) compared to those with intact cognition (1.7 hours).

**Conclusions:**

Dementia and cognitive impairment, whether diagnosed or undiagnosed by the health system, were associated with increased daily care hours among caregivers of older adults seeking emergency care. Older adults with undiagnosed cognitive impairment required nearly ten hours of care daily, suggesting that improved diagnostic screening capabilities may optimize resource allocation to support caregivers.

